# Incidence and disease burden of bronchiectasis in systemic lupus erythematosus: a nationwide population-based study in Korea

**DOI:** 10.1136/rmdopen-2025-006671

**Published:** 2026-06-22

**Authors:** Geonhui Min, Dong Eun Kye, Heajung Lee, Yeonghee Eun, Hyung Koo Kang, Hayoung Choi, Bumhee Yang, Hyun Lee

**Affiliations:** 1 Department of Biochemistry, Chungbuk National University College of Medicine, Chungbuk National University, Cheongju-si, The Republic of Korea; 2 Department of internal medicine, Chungbuk National University College of Medicine, Chungbuk National University, Cheongju-si, The Republic of Korea; 3 Department of Statistics and Data Science, Yonsei University, Seoul, The Republic of Korea; 4 Division of Rheumatology, Department of Internal Medicine, Kangbuk Samsung Hospital, Seoul, The Republic of Korea; 5 Division of Pulmonary and Critical Care Medicine, Department of Internal Medicine, Ilsan Paik Hospital, Inje University College of Medicine, Goyang, The Republic of Korea; 6 Division of Pulmonary, Allergy, and Critical Care Medicine, Department of Internal Medicine, Hallym University Kangnam Sacred Heart Hospital, Seoul, The Republic of Korea; 7 Division of Pulmonary and Critical Care Medicine, Department of Internal Medicine, Chungbuk National University Hospital, Chungbuk National University College of Medicine, Cheongju, The Republic of Korea; 8 Division of Pulmonary Medicine and Allergy, Department of Internal Medicine, Hanyang University College of Medicine, Seoul, The Republic of Korea

**Keywords:** Lupus Erythematosus, Systemic, Incidence, Epidemiology, Autoimmune Diseases

## Abstract

**Objectives:**

Pulmonary abnormalities, such as interstitial lung disease, have been relatively well studied in patients with systemic lupus erythematosus (SLE). However, it remains unclear whether the risk of bronchiectasis and its associated disease burden are increased in SLE.

**Methods:**

Using the Korean National Health Insurance Service dataset, we conducted a population-based matched cohort study involving adults aged ≥20 years diagnosed with SLE (SLE cohort) between 2002 and 2012 and a 1:4 age, sex and health insurance service-matched cohort (matched controls). Beginning 1 year after enrolment, participants were followed until the date of a bronchiectasis diagnosis, death or 31 December 2019, whichever came first.

**Results:**

During a median follow-up of 12.8 years (IQR 10.0–16.0 years), the incidence rate of bronchiectasis was higher in the SLE cohort than in the matched controls (374.8 vs 236.2/100 000 person-years, p<0.001). The SLE cohort showed a 1.46-fold (95% CI 1.22 to 1.76) increased risk of developing bronchiectasis compared with the matched cohort. Within the SLE cohort, age older than 40 years (highest adjusted HR (aHR) in those ≥70 years 25.08, 95% CI 7.62 to 82.53) and asthma (aHR 1.73, 95% CI 1.11 to 2.70) were associated with an increased risk of bronchiectasis. In contrast, obesity was inversely associated with the risk of bronchiectasis (aHR 0.56, 95% CI 0.38 to 0.82). Individuals with SLE who developed bronchiectasis had significantly higher rates of mortality, emergency department visits and hospitalisations than those who did not develop bronchiectasis.

**Conclusions:**

The risk of bronchiectasis was higher in individuals with SLE than in controls, and its development was associated with a worse disease course, higher mortality during follow-up and increased healthcare utilisation.

**WHAT IS ALREADY KNOWN ON THIS TOPIC**

- Pulmonary complications such as interstitial lung disease are well documented in systemic lupus erythematosus (SLE), but population-based evidence regarding the association between SLE and bronchiectasis remains limited.

**WHAT THIS STUDY ADDS**

- This nationwide cohort study indicates that individuals with SLE have an increased risk of developing bronchiectasis compared with matched controls.
- The association remains consistent when accounting for potential overlap with other connective tissue diseases.
- Within the SLE population, certain demographic and clinical factors are associated with higher bronchiectasis risk.
- Patients with SLE with bronchiectasis also experience increased healthcare utilisation.

**HOW THIS STUDY MIGHT AFFECT RESEARCH, PRACTICE OR POLICY**

- These findings highlight the importance of proactive respiratory monitoring in patients with SLE.
- Early recognition of bronchiectasis may facilitate timely interventions to prevent disease progression and improve long-term outcomes in this vulnerable population.

## Introduction

 Systemic lupus erythematosus (SLE) is a chronic, multisystem autoimmune disease characterised by diverse clinical manifestations and fluctuating disease activity.^1 2^ Pulmonary involvement occurs in up to 50%–70% of patients with SLE and encompasses pleuritis, pulmonary hypertension, diffuse alveolar haemorrhage and interstitial lung disease (ILD).^3 4^ Although lung parenchymal involvement in ILD in SLE has been well characterised—with documented effects on morbidity and long-term outcomes—the full spectrum of SLE-related respiratory complications remains incompletely defined.

Bronchiectasis is a chronic respiratory disease defined radiographically by irreversible bronchial dilatation and clinically by chronic cough, sputum production and recurrent respiratory infections. It is associated with persistent airway inflammation and may result in substantial morbidity and mortality.^5^ Connective tissue diseases (CTDs), such as rheumatoid arthritis (RA) and systemic sclerosis, have been shown to be associated with an elevated bronchiectasis risk.^6 7^ Furthermore, when CTDs and bronchiectasis coexist, the prognosis is worse than when either disease is presented independently.^8 9^ Although those findings suggest that systemic inflammation and immune dysregulation in CTDs might contribute to airway damage, no association between SLE and bronchiectasis has been clearly elucidated. Although several case reports and small-scale cohort studies have reported the occurrence of bronchiectasis in patients with SLE,^10^,^12^ large-scale population-based studies investigating such an association remain rare. Furthermore, it is not known whether the presence of bronchiectasis significantly affects the natural disease course of SLE. Clarifying this relationship is important to inform surveillance strategies and early interventions that could preserve lung health in patients with SLE.

To address this gap, we conducted a nationwide, population-based, matched-cohort study using the Korean National Health Insurance Service (NHIS) database. We compare the risk of incident bronchiectasis in adults with SLE with that in matched controls, identify SLE-specific risk factors for bronchiectasis and evaluate the effects of bronchiectasis on mortality and healthcare utilisation.

## Methods

### Data source and setting

This study used the Korean NHIS-National Sample Cohort (NHIS-NSC) database, a representative population-based retrospective cohort in Korea. The NHIS-NSC database has data on: (1) demographic variables (age and sex); (2) socioeconomic variables (eg, type of insurance, residential area); (3) healthcare utilisation (outpatient department visits, emergency department (ED) visits and hospitalisations); (4) health screening examination findings (eg, body mass index (BMI), smoking status and alcohol consumption) and (5) disease diagnoses based on the International Classification of Diseases, 10th Revision (ICD-10), medical treatment, procedures and surgery.^13^ Various epidemiological studies for bronchiectasis that relied extensively on NHIS data have already been published.^14^,^18^

### Study population

Between 1 January 2002 and 31 December 2012, the database contains data for 554 804 adults aged ≥20 years. After excluding missing values in variables required for cohort selection or analysis (complete-case analysis) (n=26 178) and those diagnosed with SLE between 1 January 2002 and 31 December 2002 (n=255), we identified 528 371 SLE-naïve adult participants. Among them, individuals with a diagnosis of bronchiectasis prior to enrolment (n=5090), those who developed bronchiectasis within the 1-year washout period (n=1368) and those who died during the washout period (n=750) were excluded. Consequently, 521 163 bronchiectasis-naïve adults were included in the analysis. Of them, 3368 participants were diagnosed with SLE (SLE cohort), and the remaining 517 795 had no diagnosis of SLE (non-SLE control cohort), defined as individuals who did not receive an SLE diagnosis throughout the entire follow-up period.

The index date (t0) was defined as the date of the first SLE diagnosis for the SLE cohort and the date of the earliest general health examination during 2002–2012 for the matched controls.

**Figure 1:**
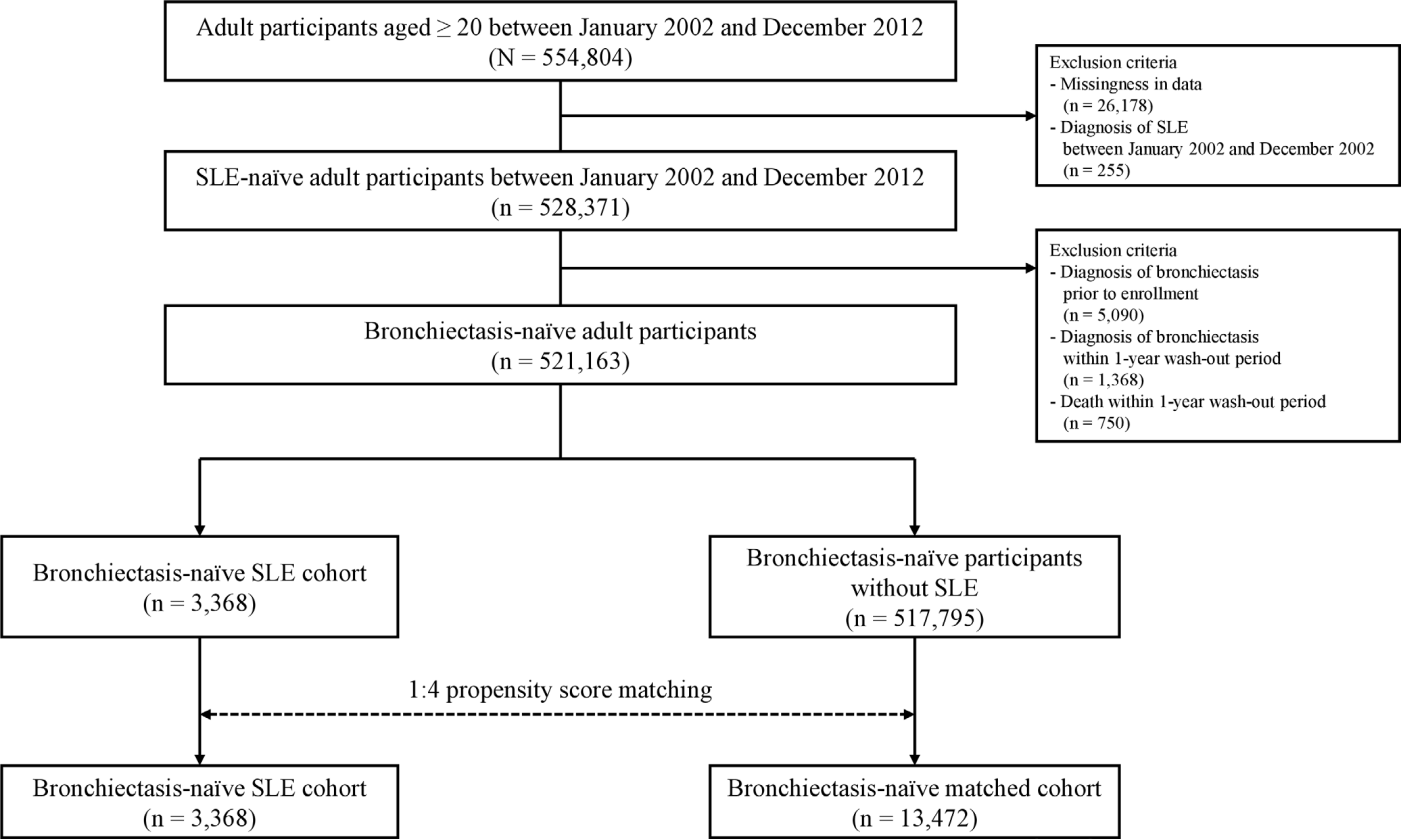
Flow chart of study participants. SLE, systemic lupus erythematosus.

To establish a matched cohort, 1:4 propensity score matching was performed based on age, sex and type of insurance, resulting in a matched cohort of 13 472 individuals without SLE. For the matched non-SLE cohort, follow-up began on the date of the earliest general health examination during 2002–2012. Participants were followed up until death, a bronchiectasis diagnosis or 31 December 2019, whichever occurred first (figure 1).

### Exposure

SLE was defined as the presence of a major or minor diagnostic code associated with ICD-10 code M32 and a special registration code (V136) provided by the Rare Intractable Diseases (RID) programme of the NHIS.^3^

The RID programme of the NHIS in Korea provides significant financial support for patients with SLE, covering up to 90% of their medical costs. Registration requires a strict government review, ensuring that only eligible patients are approved. In addition, specialists in internal medicine must assign the official diagnostic code before enrolment, which guarantees clinical accuracy. This dual system of administrative screening and specialist verification minimises misdiagnosis and makes the programme highly reliable. As a result, the RID programme reduces the economic burden on patients and generates trustworthy data for national research and policy development.

### Outcomes

Our major study outcome was the risk of bronchiectasis in individuals with SLE versus controls. Bronchiectasis was defined using the ICD-10 diagnosis code J47.^19^

Our minor study outcomes were to identify risk factors for bronchiectasis in the SLE cohort and compare the disease burden of individuals with SLE according to the presence or absence of bronchiectasis.

As the disease burden, we considered healthcare utilisation (ED visits and hospital admissions) and mortality. All-cause and respiratory-related death were evaluated using data from Statistics Korea (an initiative of the Ministry of Strategy and Finance, Republic of Korea).^20^ Respiratory-related healthcare utilisation was defined as ED visits or hospitalisations with ICD-10 codes for any respiratory system disease (J00–J99) as a major or minor diagnosis. Respiratory disease-related mortality was defined as death with a primary diagnosis coded under ICD-10 codes J00–J99.^21^

### Covariates

The following covariates were considered in the analysis: demographic factors (age, sex, income level and insurance type), lifestyle factors (smoking status, alcohol consumption and physical activity), comorbidities, the Charlson Comorbidity Index (CCI) and medication history. Baseline covariates were defined at the index date. Comorbidities, CCI and medication history were assessed using claims data prior to the index date to ensure appropriate temporal ordering.

BMI was calculated as weight (kg) divided by the square of height (m) and classified according to the recommendations for Asians as follows: underweight (<18.5 kg/m^2^), normal (18.5–22.9 kg/m^2^), overweight (23.0–24.9 kg/m^2^) and obese (≥25.0 kg/m^2^). Smoking status was classified as people who had never smoked, people who previously smoked, and people who currently smoke.^22^ Economic status was classified as high income (80th–100th percentile), middle income (40th–80th percentile) and low income (<40th percentile). The type of insurance was classified as self-employed health insurance or employee health insurance. Alcohol consumption and physical activity were categorised based on the number of days per week of alcohol consumption and physical activity participation, respectively.^23^ CCI was calculated by observing comorbidities during the preceding year. Medication use was assessed for systemic corticosteroids and immunomodulators (azathioprine, mercaptopurine, ciclosporin, tacrolimus and methotrexate). Systemic corticosteroid use was defined as prescriptions for ≥2 consecutive weeks within 1 year prior to the index date (t0) and immunomodulator use was defined based on prescription records within the same period.

Comorbidities were identified based on the following ICD-10 codes: asthma (J45–J46)^24^,^26^; chronic obstructive pulmonary disease (J42–J44, except J43.0 (unilateral emphysema))^27^,^30^; cerebrovascular disease (I60–I69); cardiovascular disease, including hypertension (I10–I15); angina or myocardial infarction (I20–I25); heart failure (I43, I50, I09.9, I11.0, I25.5, I13.0, I13.2, I42.0, I42.5–I42.9 and P29.0); diabetes mellitus (E10–E14)^31^,^34^ and CTDs other than SLE (M05–M06, M33–M34, M315, M351, M353 and M360).^7 8 35^

### Statistical analysis

Categorical variables are presented as numbers (percentages), and we used the χ^2^ test to compare groups. Continuous variables are presented as means (SD) or medians (IQRs), and we used the t-test or Mann-Whitney U test to compare groups.

We used the incidence rate of bronchiectasis, which is calculated as the number of incident events per 100 000 person-years (PY), to compare the SLE and matched cohorts. The cumulative incidence of bronchiectasis between the SLE and matched cohorts was compared using a cumulative incidence curve and Gray’s test. Univariable and multivariable Cox proportional hazards regression analyses were conducted to estimate the HR and corresponding 95% CI for incident bronchiectasis in the SLE cohort, compared with the matched cohort. Model 1 was a crude model. Model 2 was adjusted for household income, BMI, smoking status, alcohol consumption, physical activity, comorbidities (asthma, chronic obstructive pulmonary disease, cerebrovascular disease, hypertension, angina or myocardial infarction, congestive heart failure, diabetes mellitus and CTDs), CCI and medications.

Covariates included in model 2 were selected based on a combination of clinical relevance, prior epidemiological evidence on bronchiectasis risk factors and associations observed in our data. Variables such as age and CCI, which showed significant associations in univariable analyses, were included, whereas variables like sex and household income, although not statistically significant, were included based on their established relevance in prior studies.^67 36^,^38^ Variables that could plausibly lie on the causal pathway between SLE and bronchiectasis were not adjusted to avoid bias from controlling for mediators. All selected covariates were retained in the model regardless of their statistical significance.

The numbers of ED visits and hospitalisations divided by the sum of the follow-up duration (per 100 000 PY) were defined as the incidence of ED visits and hospitalisations, respectively. All tests were two-sided and a p value <0.05 was considered statistically significant. All statistical analyses were performed using SAS software V.9.4 (SAS Institute, Cary, North Carolina, USA) and R V.4.2.3 (R Foundation for Statistical Computing, Vienna, Austria).

## Results

### Baseline characteristics

The baseline characteristics of the individuals (age, sex and type of insurance) exhibited a well-balanced distribution between the two cohorts (table 1). The SLE cohort had a higher rate of people with high household income (34.1% vs 30.9%, p<0.001) and people who did not smoke (78.4% vs 77.7%, p=0.022) than the matched cohort. Additionally, the SLE cohort showed a higher prevalence of hypertension (16.2% vs 13.8%, p<0.001), angina or myocardial infarction (4.7% vs 3.8%, p=0.018) and CTDs (12.1% vs 4.1%, p<0.001). A CCI score ≥2 was also higher in the SLE cohort (22.0% vs 16.8%, p<0.001). Systemic corticosteroids (8.9% vs 5.4%, p<0.001) and immunomodulators (3.8% vs 0.6%, p<0.001) were used significantly more frequently in the SLE cohort than in the matched cohort.

**Table 1:** Baseline characteristics of the study patients

Variable	Total (n=16 840)	SLE cohort (n=3368)	Matched cohort (n=13 472)	P value
Age (years)				>0.999
20–29	2565 (15.2)	513 (15.2)	2052 (15.2)	
30–39	2295 (13.6)	459 (13.6)	1836 (13.6)	
40–49	5980 (35.5)	1196 (35.5)	4784 (35.5)	
50–59	3565 (21.2)	713 (21.2)	2852 (21.2)	
60–69	1940 (11.5)	388 (11.5)	1552 (11.5)	
≥70	495 (2.9)	99 (2.9)	396 (2.9)	
Sex				>0.999
Male	4590 (27.3)	918 (27.3)	3672 (27.3)	
Female	12 250 (72.7)	2450 (72.7)	9800 (72.7)	
Type of insurance				>0.999
Self-employed health insurance	5720 (34.0)	1144 (34.0)	4576 (34.0)	
Employee health insurance	11 120 (66.0)	2224 (66.0)	8896 (66.0)	
Household income				<0.001
Low	3348 (19.9)	579 (17.2)	2769 (20.6)	
Middle	8177 (48.6)	1642 (48.8)	6535 (48.5)	
High	5315 (31.6)	1147 (34.1)	4168 (30.9)	
Body mass index				0.356
Underweight	751 (4.5)	143 (4.3)	608 (4.5)	
Normal	7378 (43.8)	1519 (45.1)	5859 (43.5)	
Overweight	3724 (22.1)	739 (21.9)	2985 (22.2)	
Obese	4987 (29.6)	967 (28.7)	4020 (29.8)	
Smoking status				0.022
Never smoker	13 106 (77.8)	2642 (78.4)	10 464 (77.7)	
Ex-smoker	1017 (6.0)	226 (6.7)	791 (5.9)	
Current smoker	2717 (16.1)	500 (14.9)	2217 (16.5)	
Alcohol consumption (days/week)				0.291
None	10 277 (61.0)	2091 (62.1)	8186 (60.8)	
1–4	6165 (36.6)	1205 (35.8)	4960 (36.8)	
≥5	398 (2.4)	72 (2.1)	326 (2.4)	
Physical activity (days/week)				0.004
None	10 571 (62.8)	2031 (60.3)	8540 (63.4)	
1–4	5103 (30.3)	1085 (32.2)	4018 (29.8)	
≥5	1166 (6.9)	252 (7.5)	914 (6.8)	
Comorbidities				
Pulmonary comorbidities				
Asthma	1699 (10.1)	337 (10.0)	1362 (10.1)	0.858
Chronic obstructive pulmonary disease	609 (3.6)	136 (4.0)	473 (3.5)	0.143
Cerebrovascular disease	371 (2.2)	79 (2.4)	292 (2.2)	0.529
Cardiovascular disease				
Hypertension	2406 (14.3)	546 (16.2)	1860 (13.8)	<0.001
Angina or myocardial infarction	665 (4.0)	157 (4.7)	508 (3.8)	0.018
Congestive heart failure	230 (1.4)	46 (1.4)	184 (1.4)	>0.999
Diabetes mellitus	1353 (8.0)	292 (8.7)	1061 (7.9)	0.129
Connective tissue diseases	957 (5.7)	406 (12.1)	551 (4.1)	<0.001
CCI				<0.001
0–1	13 835 (82.2)	2626 (78.0)	11 209 (83.2)	
≥2	3005 (17.8)	742 (22.0)	2263 (16.8)	
Medication				
Systemic corticosteroid use	1029 (6.1)	300 (8.9)	729 (5.4)	<0.001
Immunomodulator	208 (1.2)	129 (3.8)	79 (0.6)	<0.001

Data are presented as number (%).

CCI, Charlson Comorbidity Index; SLE, systemic lupus erythematosus.

### Risk of bronchiectasis in individuals with SLE

**Figure 2:**
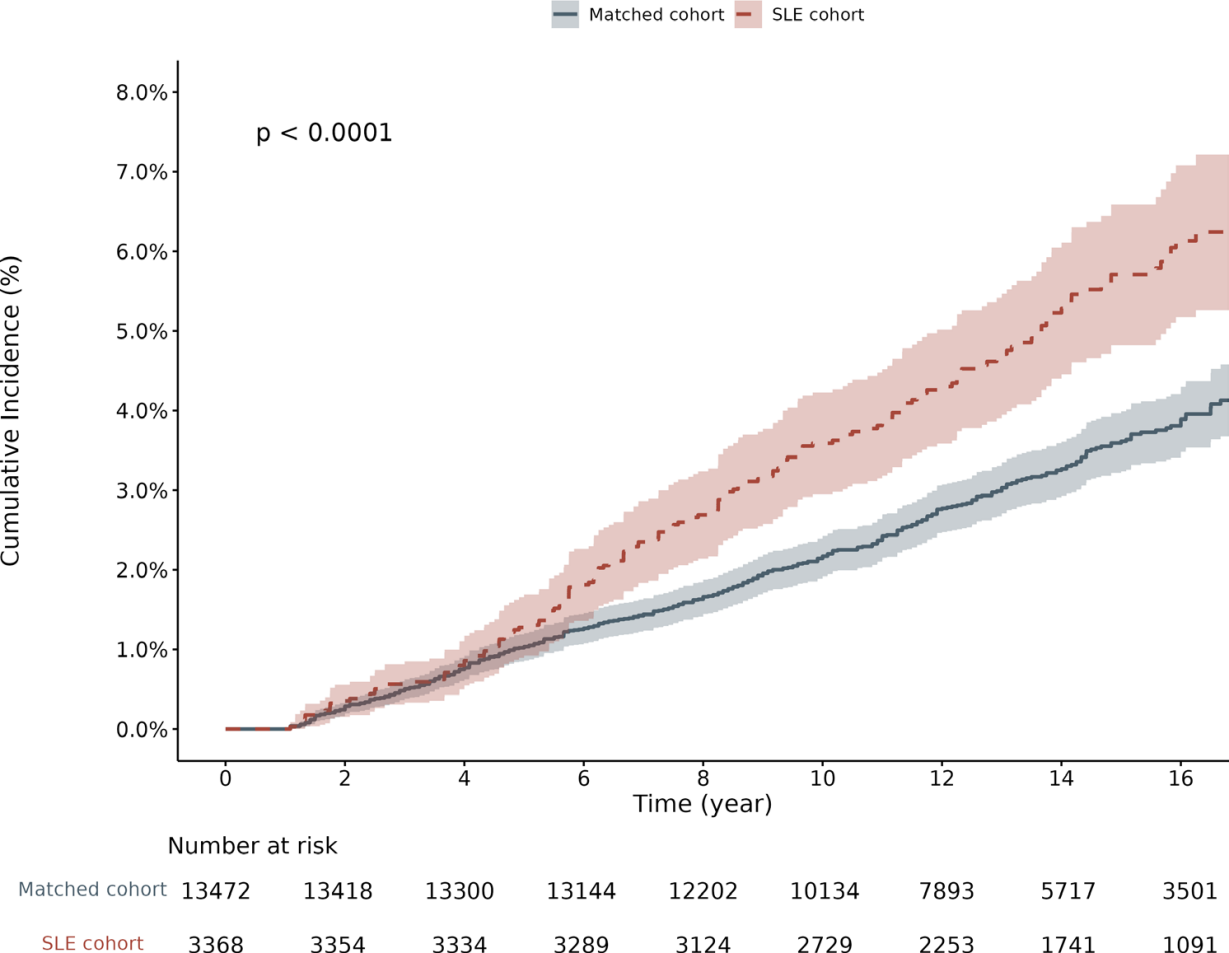
Cumulative incidence for bronchiectasis in the SLE and matched cohorts. SLE, systemic lupus erythematosus.

During a median follow-up duration of 12.8 years (IQR 10.0–16.0 years), the incidence rate of bronchiectasis was higher in the SLE cohort than in the matched cohort (374.76 vs 236.15 per 100 000 PY; p<0.001) (table 2). The cumulative incidence plot showed similar results (Gray’s test, p<0.001) (figure 2).

**Table 2:** Incidence rates and HR for bronchiectasis in the SLE and matched cohorts according to sociodemographic and lifestyle risk factors

Variable	Number of patients	Cases	Incidence rate (/100 000 PY)	Model 1		Model 2	
				HR (95% CI)	P value	HR (95% CI)	P value
Overall							
Matched cohort	13 472	402	236.15	1 (ref)		1 (ref)	
SLE cohort	3368	168	374.76	1.57 (1.32 to 1.89)	<0.001	1.46 (1.22 to 1.76)	<0.001
Age (years)							
20–39							
Matched cohort	3888	36	72.13	1 (ref)		1 (ref)	
SLE cohort	972	14	105.04	1.48 (0.80 to 2.75)	0.212	1.60 (0.86 to 2.98)	0.139
40–59							
Matched cohort	7636	246	254.97	1 (ref)		1 (ref)	
SLE cohort	1909	100	393.39	1.52 (1.21 to 1.92)	<0.001	1.47 (1.16 to 1.86)	0.001
≥60							
Matched cohort	1948	120	503.45	1 (ref)		1 (ref)	
SLE cohort	487	54	888.25	1.76 (1.28 to 2.43)	<0.001	1.58 (1.14 to 2.21)	0.007
P for interaction					0.742		0.725
Sex							
Male							
Matched cohort	3672	104	221.70			1 (ref)	
SLE cohort	918	46	364.82	1.64 (1.16 to 2.32)	0.006	1.41 (0.98 to 2.02)	0.061
Female							
Matched cohort	9800	298	241.65			1 (ref)	
SLE cohort	2450	122	378.66	1.55 (1.26 to 1.92)	<0.001	1.47 (1.18 to 1.82)	<0.001
P for interaction					0.825		0.712
Type of insurance							
Self-employed							
Matched cohort	4576	161	286.18	1 (ref)		1 (ref)	
SLE cohort	1144	66	450.26	1.55 (1.17 to 2.07)	0.003	1.52 (1.14 to 2.04)	0.005
Employee							
Matched cohort	8896	241	211.45	1 (ref)		1 (ref)	
SLE cohort	2224	102	338.09	1.59 (1.26 to 2.01)	<0.001	1.45 (1.14 to 1.84)	0.002
P for interaction					0.942		0.804
Household income							
Low							
Matched cohort	2769	72	205.14	1 (ref)		1 (ref)	
SLE cohort	579	23	299.06	1.45 (0.90 to 2.31)	0.124	1.31 (0.80 to 2.14)	0.287
Middle							
Matched cohort	6535	179	218.01	1 (ref)		1 (ref)	
SLE cohort	1642	85	390.89	1.78 (1.38 to 2.31)	<0.001	1.77 (1.36 to 2.30)	<0.001
High							
Matched cohort	4168	151	284.77	1 (ref)		1 (ref)	
SLE cohort	1147	60	389.81	1.36 (1.01 to 1.83)	0.047	1.22 (0.90 to 1.66)	0.201
P for interaction					0.382		0.280
Body mass index							
Underweight							
Matched cohort	608	15	199.92	1 (ref)		1 (ref)	
SLE cohort	143	8	431.01	2.15 (0.91 to 5.07)	0.081	1.98 (0.79 to 4.96)	0.145
Normal							
Matched cohort	5859	171	232.18	1 (ref)		1 (ref)	
SLE cohort	1519	82	411.52	1.76 (1.36 to 2.30)	<0.001	1.57 (1.20 to 2.06)	0.001
Overweight							
Matched cohort	2985	97	254.01	1 (ref)		1 (ref)	
SLE cohort	739	35	347.78	1.35 (0.92 to 1.99)	0.128	1.36 (0.92 to 2.02)	0.123
Obese							
Matched cohort	4020	119	233.83	1 (ref)		1 (ref)	
SLE cohort	967	43	331.22	1.41 (0.99 to 1.99)	0.056	1.34 (0.94 to 1.91)	0.102
P for interaction					0.536		0.725
Smoking status							
Never smoker							
Matched cohort	10 464	320	240.57	1 (ref)		1 (ref)	
SLE cohort	2642	131	371.89	1.53 (1.25 to 1.88)	<0.001	1.44 (1.17 to 1.78)	<0.001
Ex-smoker							
Matched cohort	791	31	323.92	1 (ref)		1 (ref)	
SLE cohort	226	15	510.32	1.59 (0.86 to 2.94)	0.142	1.41 (0.72 to 2.75)	0.316
Current smoker							
Matched cohort	2217	51	184.49	1 (ref)		1 (ref)	
SLE cohort	500	22	330.17	1.77 (1.07 to 2.91)	0.026	1.67 (1.00 to 2.80)	0.052
P for interaction					0.872		0.847
Alcohol consumption (days/week)							
None							
Matched cohort	8186	283	273.12	1 (ref)		1 (ref)	
SLE cohort	2091	114	410.80	1.49 (1.20 to 1.86)	<0.001	1.39 (1.11 to 1.74)	0.004
1–4							
Matched cohort	4960	110	175.25	1 (ref)		1 (ref)	
SLE cohort	1205	48	296.42	1.67 (1.19 to 2.35)	0.003	1.57 (1.11 to 2.21)	0.011
≥5							
Matched cohort	326	9	233.89	1 (ref)		1 (ref)	
SLE cohort	72	6	678.84	2.90 (1.03 to 8.16)	0.043	3.96 (1.24 to 12.71)	0.021
P for interaction					0.426		0.408
Physical activity (days/week)							
None							
Matched cohort	8540	253	237.66	1 (ref)		1 (ref)	
SLE cohort	2031	96	360.72	1.51 (1.20 to 1.91)	<0.001	1.45 (1.14 to 1.84)	0.002
1–4							
Matched cohort	4018	110	212.13	1 (ref)		1 (ref)	
SLE cohort	1085	50	337.62	1.57 (1.12 to 2.19)	0.008	1.36 (0.96 to 1.92)	0.085
≥5							
Matched cohort	914	39	327.19	1 (ref)		1 (ref)	
SLE cohort	252	22	646.06	1.97 (1.16 to 3.31)	0.011	1.82 (1.05 to 3.14)	0.032
P for interaction					0.667		0.760

Model 1 is the crude model.

Model 2 is adjusted for household income, body mass index, smoking status, alcohol consumption, physical activity, comorbidities, CCI and medications.

CCI, Charlson Comorbidity Index; ref, reference; SLE, systemic lupus erythematosus.

Even after adjusting for confounders, the SLE cohort showed a 1.46-fold (95% CI 1.22 to 1.76) higher risk of developing bronchiectasis than the control cohort. Age, sex, type of insurance, household income, BMI, smoking status, alcohol consumption and physical activity did not affect the association between SLE and the risk of bronchiectasis (p value for interaction >0.05 for all in model 2). In a sensitivity analysis excluding individuals with other connective tissue diseases, the association remained robust (aHR 1.53, 95% CI 1.26 to 1.86; online supplemental table S1).

### Factors associated with incident bronchiectasis in individuals with SLE

The factors associated with incident bronchiectasis in the SLE cohort are shown in table 3. In the multivariable analyses, increasing age (the highest adjusted HR was 25.08 (95% CI 7.62 to 82.53) for those ≥70 years) and asthma (adjusted HR 1.73 (95% CI 1.11 to 2.70)) were significant factors associated with an elevated risk of incident bronchiectasis. On the other hand, obesity (adjusted HR 0.56 (95% CI 0.38 to 0.82)) decreased the risk of incident bronchiectasis.

**Table 3:** Risk factors for bronchiectasis in patients with SLE

Variable	Model 1		Model 2	
	HR (95% CI)	P value	HR (95% CI)	P value
Age (years)				
20–29	1 (ref)		1 (ref)	
30–39	2.70 (0.85 to 8.62)	0.093	3.05 (0.95 to 9.80)	0.061
40–49	5.21 (1.88 to 14.45)	0.002	6.19 (2.18 to 17.61)	<0.001
50–59	9.50 (3.43 to 26.25)	<0.001	11.81 (4.13 to 33.79)	<0.001
60–69	14.24 (5.10 to 39.76)	<0.001	17.64 (6.02 to 51.69)	<0.001
≥70	21.53 (7.02 to 66.07)	<0.001	25.08 (7.62 to 82.53)	<0.001
Sex				
Male	1 (ref)		1 (ref)	
Female	1.04 (0.74 to 1.46)	0.831	1.15 (0.73 to 1.82)	0.549
Type of insurance				
Self-employed health insurance	1 (ref)		1 (ref)	
Employee health insurance	0.75 (0.55 to 1.02)	0.068	1.08 (0.78 to 1.49)	0.654
Household income				
Low	1 (ref)		1 (ref)	
Middle	1.31 (0.83 to 2.08)	0.246	1.38 (0.87 to 2.21)	0.174
High	1.31 (0.81 to 2.12)	0.270	1.04 (0.64 to 1.70)	0.874
Body mass index				
Underweight	1.05 (0.51 to 2.18)	0.890	1.73 (0.82 to 3.64)	0.149
Normal	1 (ref)		1 (ref)	
Overweight	0.84 (0.57 to 1.25)	0.391	0.69 (0.46 to 1.03)	0.070
Obese	0.80 (0.55 to 1.16)	0.240	0.56 (0.38 to 0.82)	0.003
Smoking status				
Never smoker	1 (ref)		1 (ref)	
Ex-smoker	1.38 (0.81 to 2.36)	0.238	1.63 (0.87 to 3.04)	0.125
Current smoker	0.89 (0.57 to 1.40)	0.611	1.13 (0.66 to 1.93)	0.656
Alcohol consumption (days/week)				
None	1 (ref)		1 (ref)	
1–4	0.72 (0.52 to 1.01)	0.058	0.99 (0.68 to 1.45)	0.961
≥5	1.67 (0.74 to 3.80)	0.220	1.35 (0.56 to 3.26)	0.502
Physical activity (days/week)				
None	1 (ref)		1 (ref)	
1–4	0.93 (0.66 to 1.31)	0.689	1.03 (0.72 to 1.46)	0.887
≥5	1.78 (1.12 to 2.83)	0.015	1.28 (0.79 to 2.05)	0.313
Comorbidities				
Pulmonary comorbidities				
Asthma	2.52 (1.69 to 3.75)	<0.001	1.73 (1.11 to 2.70)	0.017
Chronic obstructive pulmonary disease	2.81 (1.65 to 4.78)	<0.001	1.46 (0.82 to 2.60)	0.199
Cerebrovascular disease	1.49 (0.61 to 3.62)	0.382	0.71 (0.28 to 1.81)	0.472
Cardiovascular disease				
Hypertension	1.86 (1.31 to 2.65)	<0.001	1.10 (0.72 to 1.67)	0.662
Angina or myocardial infarction	1.63 (0.88 to 3.00)	0.119	0.88 (0.46 to 1.71)	0.710
Congestive heart failure	0.48 (0.07 to 3.46)	0.470	0.21 (0.03 to 1.51)	0.120
Diabetes mellitus	1.80 (1.15 to 2.82)	0.011	1.02 (0.63 to 1.66)	0.946
Connective tissue diseases	1.51 (0.98 to 2.31)	0.059	0.94 (0.55 to 1.60)	0.813
CCI				
0–1	1 (ref)		1 (ref)	
≥2	2.07 (1.49 to 2.88)	<0.001	1.27 (0.83 to 1.96)	0.277
Medication				
Systemic corticosteroid use	1.45 (0.90 to 2.34)	0.128	1.05 (0.63 to 1.75)	0.868
Immunomodulator	1.51 (0.74 to 3.08)	0.254	1.09 (0.48 to 2.50)	0.841
				

Model 1 is the crude model.

Model 2 is adjusted for household income, body mass index, smoking status, alcohol consumption, physical activity, comorbidities, CCI and medications.

CCI, Charlson Comorbidity Index; ref, reference; SLE, systemic lupus erythematosus.

### Mortality and healthcare utilisation

**Figure 3:**
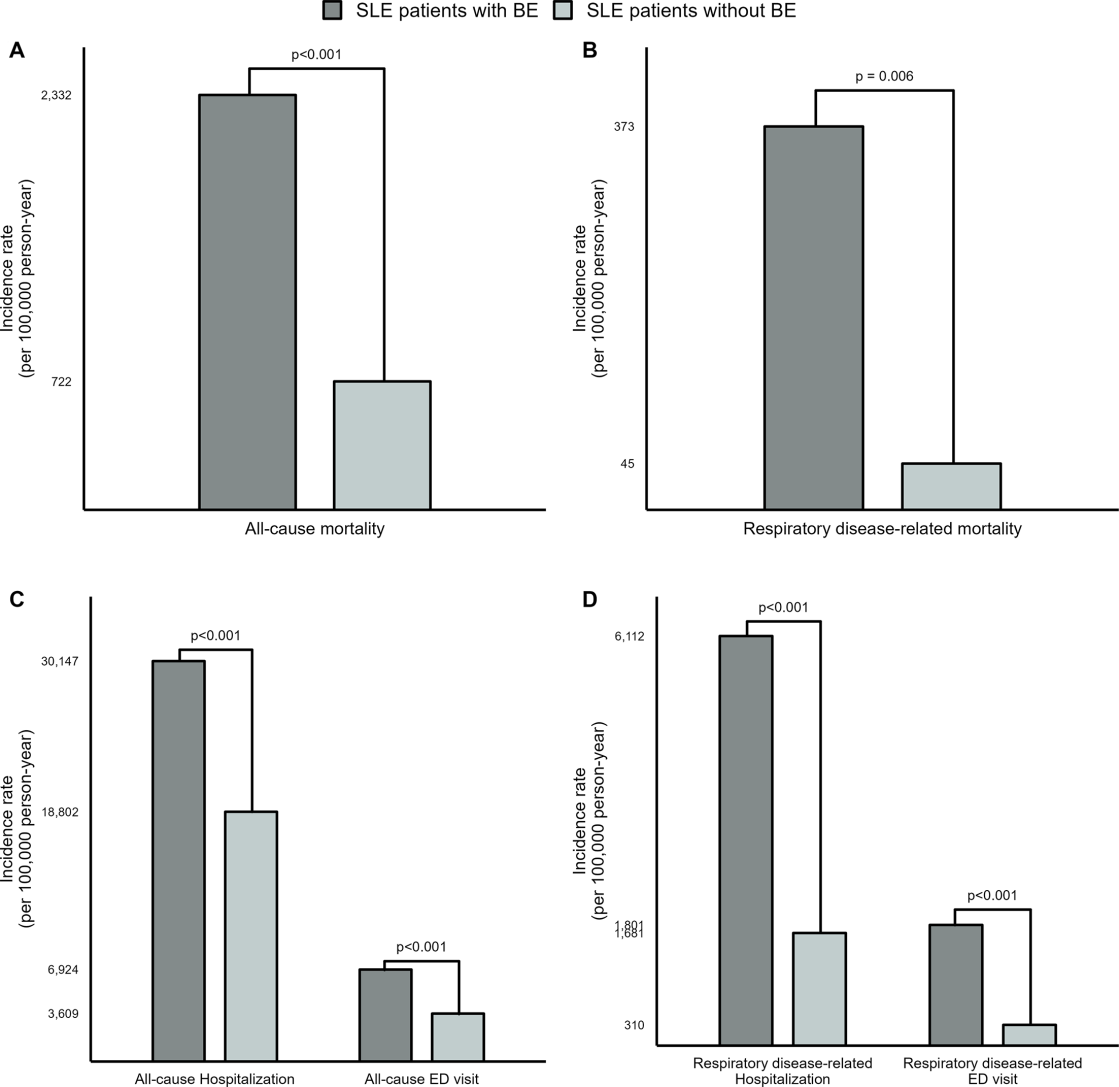
Comparison of all-cause mortality, ED visits and hospitalisations during follow-up between individuals with SLE and BE and individuals with SLE without BE. (**A**) All-cause mortality; (**B**) respiratory disease-related mortality; (**C**) all-cause ED visits and hospitalisations; (**D**) respiratory disease-related ED visits and hospitalisations. BE, bronchiectasis; ED, emergency department; SLE, systemic lupus erythematosus.

Individuals with both SLE and bronchiectasis had higher incidence rates of all-cause and respiratory-related mortality, hospitalisations and ED visits, compared with those with only SLE (p<0.05 for all) (figure 3).

## Discussion

This nationwide, population-based cohort study examined the association between SLE and subsequently diagnosed bronchiectasis using registry-based data. We observed that individuals with SLE had a higher incidence of clinically coded bronchiectasis compared with matched controls and that coexisting bronchiectasis was associated with increased healthcare utilisation and mortality during follow-up. These findings suggest that patients with SLE may be at increased risk of structural airway abnormalities recorded as bronchiectasis in administrative data.

The association between SLE and bronchiectasis is likely multifactorial.^1 2^ Chronic immune dysregulation in SLE is associated with increased susceptibility to respiratory infections and sustained pulmonary inflammation, which may contribute to irreversible airway damage in these patients.^3 39^ Moreover, SLE-related pulmonary manifestations can lead to structural changes in the bronchial tree, predisposing patients to the development of bronchiectasis.^4 40^ These hypotheses are supported by previous case series and small cohort studies, although until now, evidence from large population-based cohorts has been rare.^10^,^12^ To the best of our knowledge, ours is the first nationwide representative longitudinal population-based study to show that SLE is associated with a 1.46-fold higher risk of bronchiectasis.

Among the patients with SLE in this study, older age and the presence of asthma were found to increase the risk of bronchiectasis, reflecting the effects of age-related decline in immune function and chronic airway inflammation superimposed on SLE-related pulmonary damage. Furthermore, coexisting asthma could worsen compound epithelial damage and hypersecretion and thus make the local environment more susceptible to bronchiectasis. In contrast, obesity showed a protective association, which could be related to the so-called obesity paradox observed in chronic respiratory diseases, including chronic obstructive pulmonary disease and bronchiectasis. In a previous study that evaluated the association between BMI and bronchiectasis risk, BMI was negatively associated with the risk of developing bronchiectasis.^41^ It has been suggested that obesity might confer protective effects through leptin-mediated modulation of inflammatory responses or differences in treatment strategies.^42 43^

The increased mortality rate and healthcare utilisation of patients with SLE with bronchiectasis align with previous findings that patients with RA-bronchiectasis overlap experience a worse disease course than those with RA or bronchiectasis alone.^9 44 45^ Beyond simple addition, coexistence of those conditions could have synergistic negative effects by intensifying systemic and pulmonary inflammation, leading to higher disease activity and increasing infection risks. This concept could also be applicable to the overlap of SLE and bronchiectasis.

The increased risk of bronchiectasis and its negative effects on the disease course of SLE underscore the importance of proactive respiratory monitoring in patients with SLE. Given the almost 1.5-fold higher incidence of bronchiectasis in patients with SLE, clinicians should maintain a low threshold for investigating new or worsening respiratory symptoms—ideally through targeted history-taking, pulmonary function testing and high-resolution CT when indicated. The risk is particularly elevated among older adults and those with coexisting asthma, so careful monitoring of pulmonary symptoms is crucial in older patients and in those with asthma.

Early identification of bronchiectasis could allow the initiation of airway clearance strategies, tailored antimicrobial prophylaxis and immunomodulatory adjustments that could mitigate disease progression. Therefore, respiratory inflammatory symptoms, such as sputum production, in patients with SLE should never be overlooked, and efforts should be made to maintain sputum discharge and ensure airway clearance. Integrating routine respiratory risk assessment into SLE care pathways promises to reduce healthcare utilisation and improve long-term survival in this vulnerable population.

Beyond general immune dysregulation observed in many autoimmune diseases, SLE is characterised by immune complex deposition, complement activation and a prominent type I interferon signature, all of which may contribute to chronic inflammatory injury in pulmonary tissues.^46^,^49^ Neutrophil extracellular trap formation, a key pathogenic feature in SLE, may further amplify local inflammatory cascades and tissue remodelling within the airways.^50^,^53^ In addition, SLE-associated microvascular involvement and the frequent use of immunosuppressive therapies may increase susceptibility to recurrent pulmonary insults.^54 55^ While these mechanisms remain speculative in the context of our registry-based study, they provide biologically plausible pathways that warrant further investigation.

Our study has some limitations. First, there might be healthy user bias because our study was exclusively performed on people who received health screenings. Second, bronchiectasis was identified using ICD-10 diagnosis codes, so we might have missed subclinical or misclassified cases. In addition, because radiological images were not available for review, misclassification between conventional bronchiectasis and traction bronchiectasis associated with ILD cannot be completely excluded. Third, residual confounding cannot be ruled out despite adjustment for several known risk factors because certain clinical variables—such as pulmonary function test results, imaging data and specific medication adherence—were not available in the dataset. Fourth, this study was conducted exclusively in a Korean population, which could limit the generalisability of our findings to other ethnicities and healthcare systems.

In conclusion, our results suggest that SLE is an independent risk factor for the development of bronchiectasis. Furthermore, individuals with both SLE and bronchiectasis had higher rates of mortality and healthcare utilisation than those without bronchiectasis. These findings underscore the importance of early recognition and comprehensive management of bronchiectasis in patients with SLE.

## Supplementary material

10.1136/rmdopen-2025-006671 online supplemental file 1

## Data Availability

Data may be obtained from a third party and are not publicly available.
