# Supplementary material for: Incidence and disease burden of bronchiectasis in systemic lupus erythematosus: a nationwide population-based study in Korea
Source: RMD Open. 2026 Jun 22;12(2):e006671. doi: 10.1136/rmdopen-2025-006671 (PMC13289220; doi:10.1136/rmdopen-2025-006671)
Supplement: online supplemental file 1 [file rmdopen-12-2-s001.docx]

**Supplementary Table 1.** Incidence rates and HR for BE in SLE cohort and matched cohort (without connective tissue diseases)

according to sociodemographic and lifestyle risk factors

|  | | | | **Model 1** | | **Model 2** | |
| --- | --- | --- | --- | --- | --- | --- | --- |
| **Variable** | **Number of patients** | **Cases** | **Incidence rate**  **(/100,000 PY)** | **HR (95% CI)** | **p-value** | **HR (95% CI)** | **p-value** |
| Overall |  |  |  |  |  |  |  |
| Matched cohort | 12,921 | 370 | 225.70 | 1 (Ref) |  | 1 (Ref) |  |
| SLE cohort | 2,962 | 143 | 356.59 | 1.56 (1.29,1.90) | <0.001 | 1.53 (1.26,1.86) | <0.001 |
| Age (years) |  |  |  |  |  |  |  |
| 20-40 |  |  |  |  |  |  |  |
| Matched cohort | 3,854 | 36 | 72.69 | 1 (Ref) |  | 1 (Ref) |  |
| SLE cohort | 900 | 14 | 112.22 | 1.57 (0.85,2.92) | 0.151 | 1.60 (0.86,2.98) | 0.139 |
| 40-60 |  |  |  |  |  |  |  |
| Matched cohort | 7,277 | 230 | 249.20 | 1 (Ref) |  | 1 (Ref) |  |
| SLE cohort | 1,664 | 88 | 390.24 | 1.54 (1.21,1.97) | <0.001 | 1.52 (1.19,1.94) | <0.001 |
| ≥ 60 |  |  |  |  |  |  |  |
| Matched cohort | 1,790 | 104 | 470.30 | 1 (Ref) |  | 1 (Ref) |  |
| SLE cohort | 398 | 41 | 807.56 | 1.71 (1.19,2.45) | 0.004 | 1.65 (1.15,2.38) | 0.007 |
| P for interaction |  |  |  |  | 0.897 |  | 0.859 |
| Sex |  |  |  |  |  |  |  |
| Male |  |  |  |  |  |  |  |
| Matched cohort | 3,609 | 97 | 209.79 | 1 (Ref) |  | **1 (Ref)** |  |
| SLE cohort | 869 | 40 | 332.62 | 1.57 (1.09,2.27) | 0.016 | **1.53 (1.06,2.22)** | **0.025** |
| Female |  |  |  |  |  |  |  |
| Matched cohort | 9,312 | 273 | 231.95 | 1 (Ref) |  | 1 (Ref) |  |
| SLE cohort | 2,093 | 103 | 366.86 | 1.56 (1.25,1.96) | <0.001 | 1.53 (1.22,1.93) | <0.001 |
| P for interaction |  |  |  |  | 0.997 |  | 0.988 |
| Type of insurance |  |  |  |  |  |  |  |
| Self-employed |  |  |  |  |  |  |  |
| Matched cohort | 4,366 | 152 | 282.28 | 1 (Ref) |  | 1 (Ref) |  |
| SLE cohort | 990 | 58 | 450.82 | 1.57 (1.16,2.13) | 0.004 | 1.55 (1.14,2.10) | 0.005 |
| Employee |  |  |  |  |  |  |  |
| Matched cohort | 8,555 | 218 | 198.03 | 1 (Ref) |  | 1 (Ref) |  |
| SLE cohort | 1,972 | 85 | 312.08 | 1.56 (1.22,2.01) | <0.001 | 1.55 (1.20,1.99) | <0.001 |
| P for interaction |  |  |  |  | 0.932 |  | 0.916 |
| Household income |  |  |  |  |  |  |  |
| Low |  |  |  |  |  |  |  |
| Matched cohort | 2,654 | 63 | 186.46 | 1 (Ref) |  | 1 (Ref) |  |
| SLE cohort | 494 | 20 | 298.70 | 1.59 (0.96,2.63) | 0.071 | 1.63 (0.98,2.71) | 0.058 |
| Middle |  |  |  |  |  |  |  |
| Matched cohort | 6,309 | 171 | 215.17 | 1 (Ref) |  | 1 (Ref) |  |
| SLE cohort | 1,475 | 73 | 368.79 | 1.70 (1.29,2.24) | <0.001 | 1.72 (1.31,2.27) | <0.001 |
| High |  |  |  |  |  |  |  |
| Matched cohort | 3,958 | 136 | 268.38 | 1 (Ref) |  | 1 (Ref) |  |
| SLE cohort | 993 | 50 | 367.32 | 1.35 (0.97,1.87) | 0.071 | 1.31 (0.95,1.82) | 0.104 |
| P for interaction |  |  |  |  | 0.574 |  | 0.486 |
| Body mass index |  |  |  |  |  |  |  |
| Underweight |  |  |  |  |  |  |  |
| Matched cohort | 596 | 14 | 189.21 | 1 (Ref) |  | 1 (Ref) |  |
| SLE cohort | 119 | 7 | 442.27 | 2.32 (0.93,5.74) | 0.070 | 2.15 (0.85,5.45) | 0.108 |
| Normal |  |  |  |  |  |  |  |
| Matched cohort | 5,647 | 158 | 221.81 | 1 (Ref) |  | 1 (Ref) |  |
| SLE cohort | 1,330 | 68 | 382.54 | 1.71 (1.29,2.28) | <0.001 | 1.62 (1.21,2.15) | 0.001 |
| Overweight |  |  |  |  |  |  |  |
| Matched cohort | 2,845 | 87 | 237.76 | 1 (Ref) |  | **1 (Ref)** |  |
| SLE cohort | 661 | 33 | 361.72 | 1.50 (1.00,2.23) | 0.050 | **1.57 (1.05,2.36)** | **0.028** |
| Obese |  |  |  |  |  |  |  |
| Matched cohort | 3,833 | 111 | 227.88 | 1 (Ref) |  | 1 (Ref) |  |
| SLE cohort | 852 | 35 | 301.20 | 1.31 (0.90,1.92) | 0.160 | 1.29 (0.88,1.88) | 0.199 |
| P for interaction |  |  |  |  | 0.577 |  | 0.637 |
| Smoking status |  |  |  |  |  |  |  |
| Never smoker |  |  |  |  |  |  |  |
| Matched cohort | 9,980 | 293 | 229.94 | 1 (Ref) |  | 1 (Ref) |  |
| SLE cohort | 2,286 | 112 | 360.46 | 1.55 (1.25,1.93) | <0.001 | 1.53 (1.23,1.90) | <0.001 |
| Ex-smoker |  |  |  |  |  |  |  |
| Matched cohort | 772 | 28 | 297.83 | 1 (Ref) |  | 1 (Ref) |  |
| SLE cohort | 206 | 11 | 403.34 | 1.35 (0.67,2.71) | 0.403 | 1.44 (0.71,2.94) | 0.313 |
| Current smoker |  |  |  |  |  |  |  |
| Matched cohort | 2,169 | 49 | 180.74 | 1 (Ref) |  | **1 (Ref)** |  |
| SLE cohort | 470 | 20 | 317.29 | 1.73 (1.03,2.91) | 0.039 | **1.77 (1.04,3.01)** | **0.037** |
| P for interaction |  |  |  |  | 0.838 |  | 0.907 |
| Alcohol consumption (days/week) |  |  |  |  |  |  |  |
| None |  |  |  |  |  |  |  |
| Matched cohort | 7,761 | 260 | 263.23 | 1 (Ref) |  | 1 (Ref) |  |
| SLE cohort | 1,791 | 94 | 386.99 | 1.46 (1.15,1.84) | 0.002 | 1.43 (1.13,1.81) | 0.003 |
| 1-4 |  |  |  |  |  |  |  |
| Matched cohort | 4,844 | 102 | 166.09 | 1 (Ref) |  | 1 (Ref) |  |
| SLE cohort | 1,102 | 43 | 287.39 | 1.71 (1.20,2.44) | 0.003 | 1.66 (1.16,2.38) | 0.006 |
| ≥5 |  |  |  |  |  |  |  |
| Matched cohort | 316 | 8 | 213.41 | 1 (Ref) |  | 1 (Ref) |  |
| SLE cohort | 69 | 6 | 705.93 | 3.33 (1.16,9.60) | 0.026 | 4.47 (1.39,14.39) | 0.012 |
| P for interaction |  |  |  |  | 0.282 |  | 0.283 |
| Physical activity (days/week) |  |  |  |  |  |  |  |
| None |  |  |  |  |  |  |  |
| Matched cohort | 8,175 | 233 | 227.77 | 1 (Ref) |  | 1 (Ref) |  |
| SLE cohort | 1,780 | 83 | 349.68 | 1.53 (1.19,1.96) | 0.001 | 1.51 (1.18,1.94) | 0.001 |
| 1-4 |  |  |  |  |  |  |  |
| Matched cohort | 3,878 | 100 | 198.91 | 1 (Ref) |  | 1 (Ref) |  |
| SLE cohort | 960 | 41 | 307.50 | 1.52 (1.05,2.18) | 0.025 | 1.48 (1.03,2.14) | 0.036 |
| ≥5 |  |  |  |  |  |  |  |
| Matched cohort | 868 | 37 | 325.67 | 1 (Ref) |  | 1 (Ref) |  |
| SLE cohort | 222 | 19 | 626.48 | 1.91 (1.10,3.33) | 0.022 | 1.91 (1.08,3.37) | 0.026 |
| P for interaction |  |  |  |  | 0.757 |  | 0.828 |

Model 1 is the crude model.

Model 2 is adjusted for household income, BMI, smoking status, alcohol consumption, physical activity, comorbidities, CCI, and medications.

SLE, systemic lupus erythematosus; HR, hazard ratio; CI, confidence interval; Ref, reference; CCI, Charlson Comorbidities Index
